# 

**Published:** 1846-04

**Authors:** 


					﻿APRIL AND MAY, 1846.
VOL. I.
NEW SERIES.
NO. I.
ILLINOIS AND INDIANA
MEDICAL AND SURGICAL
JOURNAL.
w
o
H
HH
W
O
t*
Pd
K
f>
W
Q
W
K
<Z2
rt
h!
n
t>
p<
M
i >
l Q
! ffl
; 2
a
! g
* w
i B
; w
1 Q
O
*
i H
i >
H
2
; o
!
i1-1
1 H
1 k!
i
a
H.
X
B
>
©
B
cnr.
EDITED BY
JAMES V. Z. BLANEY, M. D.
DANIEL BRAINARD, M. D.
WM. B. HERRICK, M. D., and
JOHN EVANS, M. D.
CHICAGO, ILLS.:
PUBLISHED BY ELLIS & FERGUS.
INDIANAPOLIS, IND.:
PUBLISHED BY C. B. DAVIS.
1 846.
AWTM TN ADVANCE.
				

